# Elevated Levels of PDGF Receptor and MDM2 as Potential Biomarkers for Formaldehyde Intoxication

**DOI:** 10.5487/TR.2008.24.1.045

**Published:** 2008-03-01

**Authors:** Min-Ho Lee, Byung-Hoon Lee, Ho-Sang Shin, Mi-Ock Lee

**Affiliations:** 17 College of Pharmacy and Bio-MAX Institute, Seoul National University, San 56-1 Sillim-dong, Gwanak-gu, Seoul, 151-742 Korea; 27 Bio-MAX Institute, Seoul National University, Seoul, 151-742 Korea; 37 Department of Environmental Education, Kongju National University, Kongju, 314-701 Korea

**Keywords:** Formaldehyde, PDGFRA, MDM2, Sick building syndrome

## Abstract

Formaldehyde has been identified as the most prevalent cause of sick building syndrome (SBS), which has become a major social problem, especially in developing urban areas. However, studies on the molecular mechanisms associated with formaldehyde toxicity have been limited, probably because it is difficult to relate the experimental results obtained from *in vitro* studies to human exposure *in vivo*. Using polymerase chain reaction-based suppression subtractive hybridization, we recently identified 27 different formaldehyde-inducible genes including platelet-derived growth factor receptor alpha gene (PDGFRA) and mouse double minute 2 (MDM2) gene which were increased significantly in both formaldehyde-exposed human trachea cells, 680. Tr, and rat tracheas. To establish a possible relationship between induction of these formaldehyde-inducible genes and symptoms of SBS, we examined expression levels of these genes in peripheral lymphocytes of residents of new apartments. Here, we report that the expression of PDGFRA and MDM2 transcripts was significantly higher in peripheral blood lymphocytes obtained from 15 residents in new buildings than in seven control individuals. Our results suggest that the elevated levels of PDGFRA and MDM2 may be associated with the formaldehyde-induced pathophysiology that is closely related with SBS, and that they deserve evaluation as potential biomarkers for formaldehyde intoxication.

## INTRODUCTION

Indoor exposure to low levels of noxious chemicals is associated with adverse health effects because people spend a lot of their time indoors (Moschandreas, 1981; Samet *et al.*, 1988). Recently, sick building syndrome (SBS), which is characterized by various symptoms such as respiratory mucosal irritation, skin reactions, unspecific hypersensitivity, mental fatigue, and headache, has become a major social problem. This is especially so in developing urban areas with many newly built apartments (Fernandez-Caldas *et al.*, 1994; Norback *et al.*, 1990). The main causes of SBS are poor indoor air quality caused by insufficient ventilation and the accumulation of noxious compounds such as formaldehyde and volatile organic compounds (Kim *et al.*, 2002; Kilburn, 2000). Of these indoor air chemical pollutants, formaldehyde has been identified as the most prevalent, probably because it is very often used for glued wood products in residential environments, such as particleboard, floor coverings, and furniture (Kilburn, 2000; Main and Hogan, 1983). Indeed, indoor formaldehyde concentrations in 46.7% of new apartments in Korea were higher than the 100 $\mu g/m^3$ that is the Japanese safety standard (NIER, 2004).

Formaldehyde has been considered as a potent respiratory and conjunctival irritant (Godish, 1990; Hendrick *et al.*, 1982). Although indoor formaldehyde exposure is hazardous to human health, studies on the molecular mechanisms associated with formaldehyde toxicity have been limited, probably because it is difficult to relate the experimental results obtained from *in vitro* studies to human exposure *in vivo*. Recently, we identified 27 different formaldehyde-inducible genes that could be associated with the biological effects of formaldehyde (Lee *et al.,* 2008). To establish a possible relationship between formaldehyde-induced gene expression and symptoms of SBS, we examined the expression levels of these genes in peripheral lymphocytes of residents of new apartments in comparison with results from control residents of old apartments. Among these genes, expression levels of those for PDGFRA and MDM2 were increased significantly in the peripheral lymphocytes of new building residents. Thus, elevated expression levels of these genes could be candidate biomarkers for formaldehyde toxicity.

## MATERIALS AND METHODS

***Blood collection from human subjects.*** Twenty-two volunteer subjects, 15 residents of new apartments and seven control residents of old apartments, were recruited from Kongju, located in Chungnam province in Korea for this study. Approval for the study was obtained from the Standing Committee for Ethics in Research on Humans at Seoul National University. Brief information sheets were distributed among the residents of newly constructed apartment to seek volunteers for this study. For measurements of indoor formaldehyde concentration, air samples were collected after an unannounced visit in May 2006. The concentration of formaldehyde in the indoor air was monitored by formaldehyde meter (W/Pump Model Z-300XP). Whole blood samples were collected from the experimental subjects into heparin-coated tubes, mixed with RNAlater® (Ambion Inc., Ausrin, TX) and stored at -20°C until use.

***Semiquantitative reverse transcription polymerase chain reaction (RT-PCR).*** Total RNA was isolated using the RiboPure-Blood® Kit (Ambion Inc.) according to the manufacturer's protocols. Isolated samples were treated with DNase I and single-stranded cDNA was synthesized from the RNA in a reaction mixture containing random hexamers and 200 U of murine Moloney leukemia virus reverse transcriptase (Invitrogen, Carlsbad, CA) (Shin *et al.*, 2003). The PCR reaction was performed with specific primers, as previously described (Lee *et al.*, 2008). The genes were analyzed under conditions in which their PCR products were amplified exponentially.

***Statistical analysis.*** Experimental values are expressed as the mean ± S.D. The significance of any difference was determined by Student's $t$ tests and expressed as a probability value. Mean differences were considered significant at $P < 0.05$.

## RESULTS

Formaldehyde is a ubiquitous chemical toxicant that probably causes SBS: adverse health effects caused by indoor air pollution in residents of newly constructed buildings. Recently, we identified 27 different formaldehyde-inducible genes that could be associated with the biological effects of formaldehyde (Lee *et al.*, 2008). To establish a possible relationship between formaldehyde-induced gene expression and symptoms of SBS, we examined the expression levels of genes for calcyclin, glutathione *S*-transferase pi, PDGFRA, MDM2 and HLA-A in peripheral lymphocytes of 15 residents of new apartments, and these were compared with results from seven control residents of old apartments. Details regarding the experimental subjects are described in Table 1. The mean indoor formaldehyde levels in the bedroom of old and new apartments were 0.027 ± 0.016 ppm and 0.311 ± 0.33 ppm, respectively; thus they were significantly higher in the new apartments (Fig. 1). The formaldehyde concentrations in new apartments ranged from 0.01 to 1.07 ppm, and nine samples (60%) exceeded 0.08 ppm, the current Japanese safety standard. To examine expression levels of formaldehyde-inducible genes, semiquantitative RT-PCR analysis was performed using mRNA obtained from whole blood samples. Intensity of the PCR bands was largely var-

**Table 1.** Demographics of study subjects[a]

| Characteristics | Residents in | | $p$-value |
|---|---|---|---|
| | Old Apartment | New Apartment | |
| Number of subjects | 7 | 15 | |
| Number of smokers | 0 (0%) | 2 (11.1%) | |
| Age (years) | 23.1 ± 3.3 | 27.7 ± 17.45 | 0.5145 |
| Duration of residence (days) | 112.2 ± 4.6 | 69.8 ± 3.7 | < 0.0001 |

[a]Data shown are the mean ± S.D.

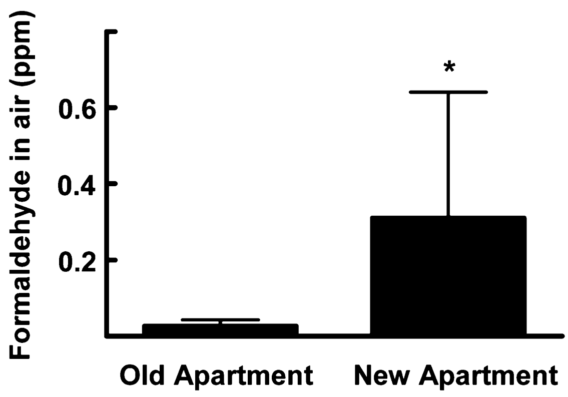

**Fig. 1.** Indoor formaldehyde levels in old and new apartments. Air samples were collected in the bedroom of 7 old apartments and 15 new apartments, and the concentration of formaldehyde was determined as described in Materials and methods. Values are represented as mean ± S.D. *, $P < 0.05$.

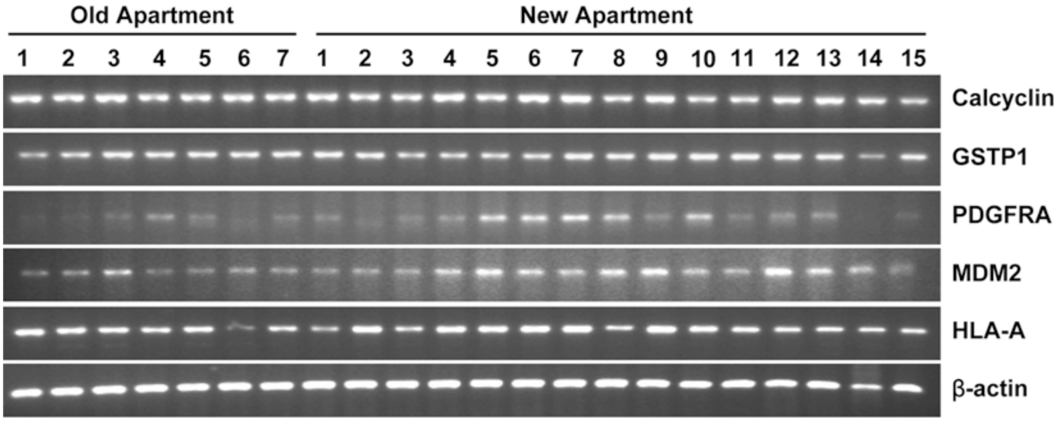

**Fig. 2.** Induction of gene transcripts in human peripheral blood monocytes obtained from residents of new buildings. Human blood samples were collected from 7 controls in old apartments and total 15 residents in new apartments as described in Table 1. The mRNA expression of the indicated gene was determined by RT-PCR with primers that are specific for each gene. Representatives of at least 3 independent experiments were shown.

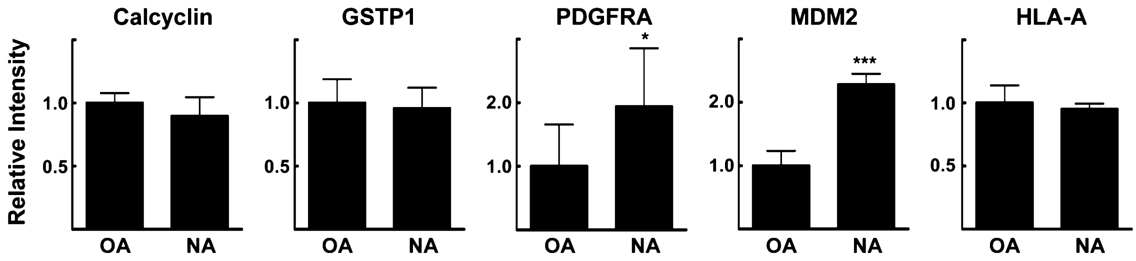

**Fig. 3.** Quantification of expression of gene transcripts in human peripheral blood monocytes obtained from residents of new buildings. The mRNA expression levels in human blood samples of 7 controls in old apartments (OA) and total 15 residents in new apartments (NA) were determined by semiquantitative RT-PCR as shown in Fig. 2. The intensity of PCR products was quantified using an image analysis system. The value was normalized to that of β-actin and expressed as relative intensity in which 1.0 represents normalized intensity for the residents of OA. Values are represented as mean ± S.D. *, $P < 0.05$ and ***, $P < 0.001$.

ied for PDGFRA and MDM2 in experimental individuals (Fig. 2). Quantification of the PCR bands showed that the expression levels of PDGFRA and MDM2 were increased approximately 1.9-fold and 2.3-fold, respectively, in the residents of new apartments to compare with the residents of old apartments (Fig. 3). However, the expression of other genes, such as those for calcyclin, GSTP1 and HLA-A, were not much altered.

## DISCUSSION

Formaldehyde is a ubiquitous chemical toxicant that probably causes SBS: adverse health effects caused by indoor air pollution in residents of newly constructed buildings. Here, we report that the expression of PDGFRA and MDM2 transcripts was significantly higher in peripheral blood lymphocytes obtained from 15 residents in new buildings than in seven control individuals. Our results suggest that the elevated levels of PDGFRA and MDM2 may be associated with the formaldehyde-induced pathophysiology that is closely related with SBS, and that they deserve evaluation as potential biomarkers for formaldehyde intoxication.

PDGFRA and PDGFRB are expressed in eosinophils and their signaling is involved in eosinophil function (Adachi *et al.*, 2006). Increased indoor concentrations of formaldehyde are associated with asthma, one of the most common respiratory diseases. These allergic symptoms induced by formaldehyde are characterized by inflammatory cell infiltration, mostly eosinophils, which are the major inflammatory cells of allergic reactions activated by respiratory mucosal damage (Casset *et al.*, 2006; Ohtsuka *et al.*, 2003). Therefore, the increase in PDGFRA level may contribute to the allergic symptoms caused by low-level exposure of air-born formaldehyde in the new apartments. The MDM2 oncoprotein is a primary regulator of the p53 tumor suppressor gene, because it controls protein level of p53 *via* ubiquitindependent proteasomal degradation. Exposure to formaldehyde increases the level of DNA damage characterized by DNA-protein crosslinks (DPC) (Heck *et al.*, 1990; Heck and Casanova, 1999; Shaham *et al.*, 2003). DPC increased mutations in p53 in peripheral blood lymphocytes of human after formaldehyde exposure, suggesting that DPC and mutation in p53 may represent steps in formaldehyde-induced carcinogenesis (Shaham *et al.*, 2003). Our results may suggest that the induction of MDM2 expression may indicate a possibility of induction of p53 expression and DNA damage after formaldehyde exposure in SBS.

In the USA, the mean concentration of indoor formaldehyde has been reported as 38 $\mu g/m^3$ (30 ppb), with a typical range of 13~350 $\mu g/m^3$ (Stenton and Hendrick, 1994). In Australia, an indoor guideline of 125 $\mu g/m^3$ (100 ppb) has been set (McPhail, 1991). Irritant effects of the upper respiratory tract are a well-established consequence of formaldehyde exposure above 150 $\mu g/m^3$ (Stenton and Hendrick, 1994). Formaldehyde exposure at levels well below 100 ppb may lead to an increased risk of allergic sensitization to common aeroallergens in children (Garrett, 1999). We found that indoor levels of formaldehyde in newly built apartments were substantially higher (310 ppb) than the present guidelines of 100 ppb in Australia and 80 ppb in Japan (McPhail, 1991). Importantly, these changes in PDGFRA and MDM2 expression patterns in residents of new buildings are consistent with our observations in this animal model in which relatively higher levels of formaldehyde were applied (3 ppm and 38 ppm) (Lee *et al.*, 2008). These results suggest that the low-level exposure to formaldehyde of new building residents may have a potential to cause the pathological symptoms of epithelial damage and allergic inflammation that was seen in following the high level exposure in rats. Taken together, indoor guidelines for formaldehyde exposure may need to be reviewed in light of these findings.

In summary, we observed that the expression levels of PDGFRA and MDM2 were increased significantly in the peripheral blood lymphocytes of the residents of newly built apartments, suggesting that these genes may be associated with the pathologies induced by formaldehyde exposure during SBS. Our findings on gene expression can be applied in follow-up programs of residents of new buildings exposed to formaldehyde to identify high risk populations and to help preventing SBS.

## ACKNOWLEDGMENTS

This study was supported by grants from the Ministry of Environment as The Eco-Technopia 21 Project and the Ministry of Education as The Brain Korea 21 Project.
